# Prevalence and factors associated with depression, anxiety and stress in Malaysia during COVID-19 pandemic: A systematic review

**DOI:** 10.1371/journal.pone.0288618

**Published:** 2023-07-20

**Authors:** Muhammad Ikhwan Mud Shukri, Halimatus Sakdiah Minhat, Norliza Ahmad, Fatin Ismail, Chandramalar Kanthavelu, Dina Nurfarahin, Wan Syahirah Wan Ghazali, Nor Afiah Mohd Zulkefli

**Affiliations:** Department of Community Health, Faculty of Medicine and Health Science, Universiti Putra Malaysia, Seremban, Selangor, Malaysia; BRAC University, BANGLADESH

## Abstract

**Background:**

The COVID-19 pandemic has had severe impacts on mental health status worldwide. Several studies have investigated the prevalence and factors associated with depression, anxiety, and stress in different countries, however, a systematic review on the research topic during COVID-19 is presently lacking in Malaysia’s context. To fill this gap, electronic databases including PubMed, Scopus, Science Direct, Sagepub, CINAHL, Psychology, and Behavioral Sciences Collection were searched for relevant studies. A total of 16 studies were included in the systematic review.

**Methods:**

To fill this gap, electronic databases including PubMed, Scopus, Science Direct, Sagepub, CINAHL, Psychology, and Behavioral Sciences Collection were searched for relevant studies. A total of 16 studies were included in the systematic review.

**Results:**

The analyses showed that the prevalence of depression, anxiety, and stress ranged from 14.3% to 81.7%, 8.0% to 81.7%, and 0.9% to 56.5% respectively. Adult populations demonstrated the highest prevalence of depression, whereas university students reported the highest prevalence of anxiety and stress. Several factors were associated with mental health conditions including age, gender, family income, and perception of COVID-19.

**Conclusion:**

Differentials in mental health screening practices call for standardised screening practices. Mental health intervention should be targeted at high-risk populations with effective risk communication.

## Introduction

The coronavirus disease (COVID-19) has become a global health emergency since its first discovery in Wuhan in November 2019. COVID-19 is caused by a novel virus known as severe acute respiratory syndrome coronavirus 2 (SARS-COV-2), which spreads primarily through respiratory droplets, contact, and airborne transmission. SARS-CoV-2 transmits in higher rate compared to other coronaviruses. It is estimated that the basic reproductive rate (*R*_0_) of SARS-COV-2 is at 2.5, comparatively higher than Middle-East respiratory syndrome coronavirus (MERS-CoV) and severe acute respiratory syndrome coronavirus (SARS-CoV) which *R*_0_ value at 0.9 and 2.4 respectively [1]. It has now spread to all countries worldwide due to its high transmissibility and was declared a pandemic by the World Health Organization (WHO) in March 2020 [2]. As of April 2022, the pandemic has infected more than 490 million people and caused more than 6.1 million deaths globally [3].

Countries worldwide implemented varying public health measures to contain the spread of the disease. These included compulsory face mask use, international travel restriction, local travel restriction, school closure and compulsory hand hygiene [4]. Preventive measures such as quarantine and isolation for infection control have been reported to impose immediate and prolonged mental health consequences in a systematic umbrella review of eight systematic reviews and meta analyses [5]. In Malaysia, strict public health measures, such as national lockdown, closure of social activities and gatherings, changes in work practice by working from home, home isolation, and travel bans were implemented during the initial phase to control and prevent the spread of COVID-19. The social and movement restriction measures have exerted negative financial, social, and health impacts. The implementation of the national lockdown through the Movement Control Order (MCO) in March 2020 has resulted in catastrophic financial loss of RM 2.4 billion daily, thereby accumulating up to RM 63 billion in two months [6]. Workers were terminated and many businesses were closed during the MCO. Police and military army were deployed across the country to implement population mobility restrictions. Although the MCO was effective in reducing the number of COVID-19 cases, the impact of these changes led to increasing mental health problems, such as depression, anxiety, and stress. Additionally, the closure of institutions, schools, non-essential businesses, and workplaces have adverse social implication at home during isolation. Uncertainty of work, fear of COVID-19, and inability to socialize with family and friends led to poorer sleep quality, insomnia, anxiety, and depression. These mental health conditions resulted in escalating numbers of suicide in Malaysia. Pre-pandemic suicide rate trend has been demonstrated to increase from 5.1 to 5.8 per 100,000 Malaysian population between 2014 and 2019 [7]. An alarming 631 suicide cases were recorded in 2020, doubling on average within the first five months of 2021 in the country [8]. Likewise, 11.1% of healthcare workers were reported to have suicidal ideation linked to several factors including depression [9]. Hence, these situations warrant effective mental health aids and interventions as the COVID-19 elimination worldwide remains uncertain.

Depression is a well-recognized primary cause of disability worldwide, and it is expected to rank first globally by 2030 [10]. Approximately 4.4% of the world population or 322 million people suffered from depression in 2015 [11]. 14.6% world population were at risk of developing depression at lifetime [12]. The overall pooled depression prevalence during COVID-19 pandemic was reported as high as 34% in general population [13]. Similar upward trends were observed in anxiety prevalence. Pre-pandemic global prevalence of anxiety escalated from 3.6% to 25% during COVID-19 pandemic [11, 14]. High prevalences of mental health problems imposed a great economic burden on every nation, and the trend of global costs of mental health problems are increasing yearly in every nation. The global cost of mental health problems was estimated to surpass 16 trillion dollars by 2030 [15].

Previous systematic reviews reported high prevalence rates of depression, anxiety, and stress across different countries [16–18]. However, these systematic reviews focused only on the prevalence [16, 18] or factors associated with depression, anxiety, and stress [17] in specific populations and countries. Findings from these systematic reviews are not generalizable to Malaysia due to different sociocultural norms, healthcare capacities, healthcare access, and public health interventions imposed for COVID-19 prevention and control. However, no systematic evaluation has been conducted to elucidate the factors influencing poor mental health status amidst the COVID-19 pandemic in Malaysia. Besides, National Health and Morbidity Survey (NHMS) 2019 revealed that 2.3% of adult Malaysian suffered from depression. Moreover, the pre-pandemic prevalence of anxiety and stress among the general population in Malaysia are lacking. Previous studies on anxiety and stress have revolved around specific populations [19, 20]. Numerous studies in Malaysia during the pandemic revealed high prevalence of depression, anxiety and stress. However, due to different screening modalities used, it limits the comparison with pre-pandemic prevalence. Hence, this systematic review is the first attempt to review studies on the prevalence and factors associated with depression, anxiety, and stress in the Malaysian population during the pandemic. These findings will provide insights for risk-based interventions for populations with poor mental health status.

## Material and methods

### Search strategy and inclusion criteria

This systematic review was conducted using the Preferred Reporting Items for Systematic Reviews and Meta-Analyses (PRISMA) 2020 reporting checklist. This review was registered on PROSPERO (ID: CRD42022337836). An in-depth literature search was conducted between 30 April 2022 and 15 May 2022 using the following electronic databases: PubMed, Scopus, Science Direct, Sagepub, CINAHL, and Psychology and Behavioral Sciences Collection. Search keywords used in all databases included 1) factors associated (factor, predictor, risk factor, protective factor, and determinant); 2) outcome (mental health, mental status, depression, anxiety, stress, and distress); 3) COVID-19 (COVID, COVID-19, pandemic; and 4) Malaysia. The search was conducted by combining the four types of keywords manually. A full-search strategy is available in the Table 1.

**Table 1:** Search strategy.

Pubmed	(((((((factor[Title/Abstract]) OR (predictor[Title/Abstract])) OR ("risk factor"[Title/Abstract])) OR ("protective factor"[Title/Abstract])) OR (determinant[Title/Abstract])) AND (((((("mental health"[Title/Abstract]) OR ("mental status"[Title/Abstract])) OR (depression[MeSH Terms])) OR (anxiety[MeSH Terms])) OR (stress[Title/Abstract])) OR (distress[Title/Abstract]))) AND (((COVID[MeSH Terms]) OR (COVID-19[MeSH Terms])) OR (pandemic[Title/Abstract]))) AND (Malaysia[Title/Abstract])
Science Direct	(factor OR predictor) AND ("mental health" OR depression OR anxiety OR stress) AND (COVID OR pandemic) AND (Malaysia)
Scopus	TITLE-ABS-KEY ((factor OR predictor OR risk AND factor OR protective AND factor OR determinant) AND (mental AND health OR mental AND status OR depression OR anxiety OR stress OR distress) AND (covid OR covid-19 OR pandemic) AND (Malaysia))
Sagepub	(factor OR predictor OR risk factor OR protective factor OR determinant) AND ("mental health" OR "mental status" OR depression OR anxiety OR stress OR distress) AND (COVID OR COVID-19 OR pandemic) AND (Malaysia)
CINAHL	(factor OR predictor OR risk factor OR protective factor OR determinant) AND ("mental health" OR "mental status" OR depression OR anxiety OR stress OR distress) AND (COVID OR COVID-19 OR pandemic) AND (Malaysia)
Psychology and Behavioral Sciences Collection	(factor OR predictor OR risk factor OR protective factor OR determinant) AND ("mental health" OR "mental status" OR depression OR anxiety OR stress OR distress) AND (COVID OR COVID-19 OR pandemic) AND (Malaysia)

Articles published until 30 April 2022 were included in this review. The inclusion criteria for articles included in this review are as follows; 1) an observational study (cross-sectional, case-control, or cohort), 2) the outcome includes the prevalence and risk factors of at least one mental health condition (depression and/or anxiety and/or stress), 3) study location in Malaysia, 4) published in the English language, 5) study duration during the COVID-19 pandemic and 6) study population as Malaysians and 7) available full text for review.

### Data collection and analysis

Each database was searched and screened by a reviewer (MI) and relevant citations based on title and abstract were imported into an electronic reference manager software. Duplicates were removed and relevant citations were imported into Microsoft Excel. A second reviewer (FI) examined the title and abstract in Microsoft Excel from an initial search for relevancy. A title list of all recruited articles was prepared; hence the articles were filtered out structurally. In the first stage, the titles and abstracts of all studies were screened according to the aforementioned inclusion criteria. In the second stage, all the articles remaining after the first stage were subjected to full-text screening accordingly. Both screenings were conducted independently by two review authors. Disagreements between the two reviewers were resolved through a discussion with a third reviewer. The AORs were obtained from multivariate analysis reported by individual studies. All information was presented in text and tables to summarize and explain the characteristics and outcomes of the studies.

### Data synthesis

The relevant data in the final articles were tabulated in Microsoft Excel according to the following points: 1) author and publication year, 2) study design, 3) sample size, 4) study population and mean age, 5) screening tools, 6) prevalence and 7) risk factors or protective factors. The prevalence was presented as absolute numbers in percentage, the confidence interval was set at 95%, and a p-value of less than 0.05 was considered significant. A meta-analysis could not be performed given the high heterogeneity of the screening tools used. The outcomes were synthesized qualitatively based on the findings from the final studies.

### Risk of bias

The final articles were evaluated for quality appraisal using Crowe Critical Appraisal Tool (CCAT). CCAT was used by two independent review authors (MI and FI) to assess the risk of bias in all final articles. The article was graded based on the percentage of the total score into high quality (≥ 75%), acceptable quality (51–74%), and poor quality (≤50%) [21].

## Results

**Fig 1:**
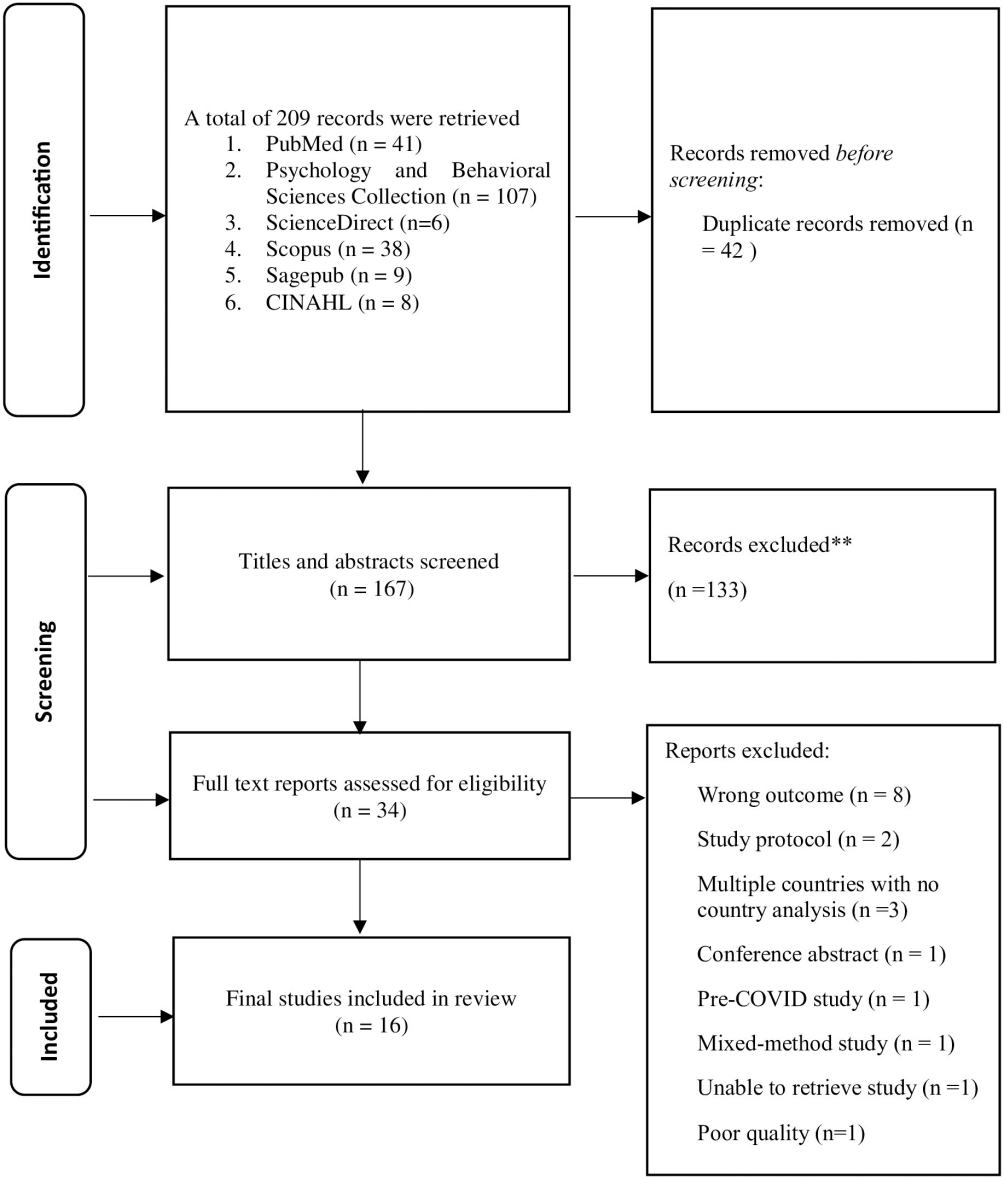
PRISMA flow chart.

The search strategy resulted in a total of 209 articles (Fig 1), which was later reduced to 167 upon removing the duplicates. Another 133 records were removed after screening the title and abstracts, resulting in 34 records. The remaining records were subjected to full-text screening, which led to the exclusion of 17 articles for the following reasons: wrong outcomes (n = 8), study protocols (n = 2), studies involving several countries with no country analysis (n = 3), and one article each as a conference abstract, pre-COVID study, and mixed-method study. Meanwhile, one article could not be retrieved despite performing an extensive search and contacting the corresponding author, journal editor, and publisher. Finally, 17 studies that fulfilled the selection criteria were included for analysis.

### Characteristics of selected studies

Table 3 outlined the characteristics of the studies included in this review. All the studies were conducted in Malaysia during the COVID-19 pandemic. All the initial discrepancies between the raters (3/17) were resolved through discussion. Overall, 13 articles were graded as high quality, three articles as acceptable quality, and one as poor quality [22] as shown in Table 2 [21]. 16 articles with high and acceptable quality were included in the review.

**Table 2:** Quality assessment of studies using Crowe Critical Analysis Tool (CCAT).

Category	Preliminary	Introduction	Design	Sampling	Data collection	Ethical matters	Result	Discussion	Total score	Percentage
Dai et al., 2020	4	5	3	3	4	5	5	4	33/40	83%
Marzo et al., 2021	5	4	3	3	3	5	4	3	28/40	70%
Li Ping Wong et al., 2021	5	5	4	3	4	5	5	5	37/40	93%
Li Ping Wong & Alias, 2021	4	5	4	3	4	5	5	5	36/40	90%
Kalok et al., 2020	4	4	3	4	4	5	4	4	32/40	80%
Moy & Ng, 2021	4	4	3	3	4	5	5	5	33/40	83%
Sundarasen et al., 2020	3	4	3	3	3	5	4	4	29/40	73%
Irfan et al., 2021	3	4	3	3	3	5	4	4	29/40	73%
Salleh et al., 2021	2	3	2	2	2	3	3	2	19/40	48%
Pang et al., 2021	5	5	4	3	4	5	5	5	36/40	90%
Ismail et al., 2021	4	4	5	3	4	5	4	4	33/40	83%
Wan Mohd Yunus et al., 2021	5	5	3	3	4	5	5	5	36/40	90%
Chew et al., 2020	5	3	3	3	3	5	5	5	32/40	80%
Narendra Kumar et al., 2022	5	4	5	4	4	5	5	5	37/40	93%
Woon et al., 2020	5	4	3	3	4	5	5	5	34/40	85%
L P Wong et al., 2021	5	4	3	3	4	5	5	5	34/40	85%
Abdul Latif et al., 2022	5	4	3	3	4	5	4	4	32/40	80%

### Study and sample characteristics

The 16 articles included in this review encompass a total of 10,928 participants, with the sample size ranging from 173 to 1,554. The participants’ mean age ranged between 21.0 (3.0) and 59.7 (11.2) years. All the studies were cross-sectional and conducted through surveys and online platforms using validated questionnaires. The participants comprised a general population (n = 4), university students, (n = 7), healthcare workers (n = 3), and patients (n = 2). The details of the included articles are summarized in Table 3.

**Table 3:** Characteristics of studies reporting depression, anxiety, and stress in Malaysia during the COVID-19 pandemic.

Author and publication year	Study design and sampling method	Data collection methods and period	Sample size	Study population and mean age	Screening tools	Outcome and prevalence	Risk factors / Protective Factors
Dai et al., 2020	Cross-sectional, stratified sampling	Online survey, May 2 to 8, 2020	669	Adults, NR	GAD-7 PHQ-9	Anxiety and depression: NR	**Risk Factors** Perceived health condition, perceived test availability, age
Marzo et al., 2021	Cross-sectional, snowball sampling	Online survey, between 15 January to 15 April 2021	1554	General population, NR	SAS PHQ-9	Anxiety: 43.6% Depression: 81.7%	**Risk Factors** Age, gender, privacy in home, exercise, victim of abuse, friends infected with COVID-19. **Protective Factors** The family’s source of income affected
Li Ping Wong et al., 2021	Cross-sectional, NR	Online survey, 12 May to 5 September 2020	1163	General adult population, 35.2±11.9	DASS-21	Depression: 21.3% Anxiety: 28.6% Stress: 12.5%	**Risk Factors** Depression: Age, student, perceived financial status, perceived health status. Anxiety: Age, gender, student, perceived health status. Stress: Age, gender, student, perceived financial status, perceived health status. **Protective Factors** Anxiety: Indian ethnicity Stress: Household income RM4001-8000
Li Ping Wong & Alias, 2021	Cross-sectional, convenience sampling	Online survey, January to April 2020	962	General population, 35.5±11.2	STAI	Anxiety: 72.1%	**Risk Factors** High perception of severity, high perceived susceptibility, fear of COVID-19, impact of COVID-19
Kalok et al., 2020	Cross-sectional study, NR	Online survey, April 2020	772	Clinical undergraduates, NR	DASS-21	Depression: 36.0% Anxiety: 44.6% Stress: 27.6%	**Risk Factors** Depression: Number of social support, perceived family support, perceived government support. Anxiety: Age, Senior students Stress: Senior student, perceived government support.
Moy & Ng, 2021	Cross-sectional, NR	Online survey, April to June 2020	367	University students, 23.0±8.0	DASS-21	Depression: 29.4% Anxiety: 51.3% Stress: 56.5%	**Risk Factors** Anxiety: Level of study Depression: Malay ethnicity **Protective Factors** Positive perception on COVID-19, Age
Sundarasen et al., 2020	Cross-sectional, NR	Online survey, 20 April to 24 May 2020	983	University students, NR	SAS	Anxiety: 8%	**Risk Factors** Gender, age, field of studies, living arrangement
Irfan et al., 2021	Cross-sectional, NR	Online survey, June-July 2020	958	Universities students, NR	GAD-7	Anxiety: 81.7%	**Risk Factors** Decreased family income, having any other diseases, COVID news time spent more than 30 minutes, infected relative or friends with COVID **Protective Factors** Having internet access
Pang et al., 2021	Cross-sectional, snowball sampling	Survey, NR	515	University students, NR	DASS-21	Depression: 46.4% Anxiety: 43.8% Stress: 26.0%	**Risk Factors** Depression: Gender and quarantine status. Anxiety and stress: Gender
Ismail et al., 2021	Cross-sectional, proportionate stratified random sampling	Survey, between 12 November and 10 December 2020	237	Medical students, 21.0±3.0	DASS-21	Anxiety: NR	**Risk Factors** Pre-clinical years **Protective Factors** Internet addiction
Wan Mohd Yunus et al., 2021	Cross-sectional, snowball sampling	Online survey, April 2020	1005	University students, NR	DASS-21	Depression: 51.5% Anxiety: 50.4% Stress: 33.9%	**Risk Factors** Anxiety: Work-family conflict, family-work conflict. Stress: Family-work conflict **Protective Factors** Happiness
Chew et al., 2020	Cross-sectional, NR	Survey, 29 April to 4 June 2020	175	Healthcare workers, 32.4±6.5	DASS-21	Depression: 14.3% Anxiety: 14.9%	**Risk Factors** Depression: Presence of symptoms
Narendra Kumar et al., 2022	Cross-sectional study, universal sampling	Survey, 1 May to 31 August 2021	173	Psychiatric healthcare workers, 36.5±8.1	HADS	Anxiety: 22.0% Depression: 16.8%	**Risk Factors** Depression: Financial hardship, short duration of service, avoidant coping. Anxiety: avoidant coping. **Protective Factors** Anxiety: married, religious coping.
Woon et al., 2020	Cross-sectional, snowball sampling	Online survey, July 2020	399	University healthcare workers, NR	DASS-21	Depression: 21.8% Anxiety: 31.6% Stress: 29.1%	**Risk Factors** Depression: Fear of COVID symptoms, quarantine as close contact. Anxiety: Single/divorced, fear of COVID symptoms, quarantine as close contact. Stress: Religion coping, fear of COVID symptoms. **Protective Factors** Depression: Having more than three children. Anxiety and stress: Friend social support.
L P Wong et al., 2021	Cross-sectional, NR	Online survey, between 23 April and 26 June 2020	631	Cancer survivors, 59.68±11.21	HADS	Anxiety: 29.0% Depression: 20.8%	**Risk Factors** Anxiety: Health status, perceived susceptibility, perceived severity, stage of cancer, ethnicity. Depression: Health status, perceived susceptibility, perceived severity, female. **Protective Factors** Income
Abdul Latif et al., 2022	Cross-sectional, NR	Survey, between October 2020 and April 2021	338	Hospital patient, undergoing COVID-19 screening in Obstetric and Gynecology Department, 33.4±7.1	DASS-21	Depression: 17.2% Anxiety: 25.1% Stress: 0.9%	**Risk Factors** Depression: Loss of income, hospital admission for surgical procedure

Note. GAD-7: Generalized AnxietyDisorder-7. PHQ-9: Patient Health Questionnaire-9. SAS: Self-Rating Anxiety Scale. DASS-21: Depression, Anxiety and Stress Scale 21. STAI: State-Trait Anxiety Inventory. HADS: Hospital Anxiety and Depression Scale. NR: Not Reported.

### Overall prevalence of depression, anxiety, and stress

Different assessment tools were used to detect mental health problems among the participants i.e., 1) DASS-21 (8 studies); 2) PHQ-9 (2 studies); 3) HADS (2 studies); 4) SAS (2 studies); 5) GAD-7 (1 study); and 6) STAI (1 study). Eleven articles reported the prevalence of depression, which ranged from 14.3% to 81.7%. The prevalence of anxiety was reported in 14 studies, with the value ranging from 8.0% to 81.7%. A wide margin of 0.9% to 56.5% was also documented in the seven studies that reported the prevalence of stress. Given the heterogeneity of assessment tools employed in the reviewed studies, a meta-analysis of the prevalence of mental health problems could not be performed. A subgroup analysis of depression, anxiety and stress across 8 studies that most utilised an identical tool i.e., DASS-21 showed that university students have the highest prevalence in all outcomes. The prevalence of depression ranges from 14.3% to 51.5%; anxiety 14.9% to 51.3% and stress 0.9% to 56.5%. However, these comparative findings should be interpreted with caution as numbers of studies among adult [23], healthcare workers [24, 25] and patients [26] are sparse.

### Group-specific prevalence of depression, anxiety, and stress

Table 4 visualized the subgroup analysis of the participants (adult population, students, healthcare workers, and patients) in the reviewed studies. Comparatively, the prevalence of mental health problems in university students was higher compared to other populations during the COVID-19 pandemic.

**Table 4:** Subgroup analysis of the prevalence of depression, anxiety, and stress.

Specific group	Depression	Anxiety	Stress
Adult population	21.3–81.7%	28.6–72.1%	12.5%
Healthcare workers	14.3–21.8%	14.9–31.6%	29.1%
University students	29.4–51.5%	8.0–81.7%	26.0–56.5%
Patients	17.2–20.8%	25.1–29.0%	0.9%

Students faced difficulties in learning through online platforms, with no adequate equipment, and limited internet coverage. Some students utilized mobile phones to attend university classes, which disrupted the effectiveness of teaching delivery. The apprehension towards the capability to sit for online university exams might have also contributed to the rising prevalence of mental health issues among students [27].

### Factors associated with depression, anxiety, and stress

The factors associated with mental health issues are complex with underlying biopsychosocial aetiologies. This systematic review identified several risks and protective factors, which were regrouped into six major categories as follows; 1) biological factors, 2) financial factors, 3) social factors, 4) academic factors, 5) psychological factors, and 6) COVID-related factors as shown in Table 5.

**Table 5:** Factors associated with depression, anxiety, and stress according to the studied population.

	Adult population	University students	Healthcare workers	Patients
**Biological factors**				
Age	[23, 28, 29]	[30–32]		
Gender	[23, 29]	[32, 33]		[34]
Ethnicity	[23]	[22, 31]		
Underlying comorbidity		[35]		[34]
Health status	[23, 28]			[34]
**Financial factors**				
Family / Household income	[23, 29]	[35]		[26, 34]
Perceived financial status	[23]		[36]	
**Social factors**				
Student	[23]			
Marital status			[25]	
Family size			[25]	
Living arrangement		[32]		
Internet access		[35]		
Hospital admission for surgical procedure				[26]
Internet addiction		[37]		
Privacy in home	[29]			
Exercise	[29]			
Social support		[30]	[25]	
Service duration			[36]	
Social conflict		[38]		
**Academic factors**				
Seniority		[30, 31, 37]		
Field of study		[32]		
**Psychological factors**				
Coping			[25, 36]	
Happiness		[38]		
**COVID-related factors**				
Presence of symptom			[24]	
COVID-related perception	[28, 39]	[31]		[34]
Exposure to news		[35]		
Infected relatives/friends	[29]	[35]		
Fear of COVID-19	[39]		[25]	
Impact of COVID-19	[39]			
Quarantine		[33]	[25]	

### Biological factors

Age, gender, ethnicity, underlying comorbidity, and health status were significantly associated with depression, anxiety, and stress among Malaysians during COVID-19. This review found that age and gender demonstrated the most reliable evidence of association with mental health issues across different populations. Younger adult population and younger university students reflected a higher level of mental health problems in comparison to older populations [23, 28–32].

Subgroup analysis revealed that among 4 specific groups of population identified in this review, adult population scored the highest prevalence of depression. In individual studies, multivariate analysis revealed that young adults are more susceptible to depression than older adults [23, 28, 29]. Adults aged 26 to 45 years were two times more likely to have depression (Adjusted Odds Ratio [AOR]: 2.07, 95% Confidence Interval [CI], 1.30–3.30) and three times more likely to suffer from stress (AOR: 3.23, 95% CI, 1.72–6.31) compared to adults age more than 45 years [25]. Young university students had higher odds of anxiety than older university students [30–32]. Younger students demonstrated higher odds of anxiety (AOR: 7.14, 95% CI, 1.46–34.88) [32] while older students were 44% less likely to experience anxiety (AOR: 0.56, 95% CI, 0.35–0.89) [30].

Female population is more vulnerable to mental health problems compared to males [23, 29, 32–34]. This finding was consistent, especially for anxiety in which females were more likely to experience the mental health issue compared to males [29, 32, 39]. Likewise, females were twice likely to suffer from depression (AOR:1.44, 95% CI, 1.32–1.62) [29], anxiety (AOR: 2.26, 95% CI, 1.25–4.10) [32], and stress (AOR: 1.83, 95% CI, 1.14–2.95) [23]. In terms of race, Indians and Chinese were reported to be less likely to experience depression and anxiety [22, 23] while Malays have a higher risk to suffer depression [31]. Having underlying comorbidities predicted risk for anxiety [34, 35]. Poor health status perceived by respondents was a risk factor for mental health conditions [23, 28, 34]. Nevertheless, the evidence on ethnicity, underlying comorbidity, and health status, are limited to the number of articles reviewed.

### Financial factors

Family income and financial status greatly affected mental health during the COVID-19 pandemic. Several studies revealed that a decrease or loss of family household income were significantly associated with mental health problem among various populations [23, 26, 29, 34, 35]. Specifically, the risk of depression increased two-fold following a decrease or complete loss of family income (AOR: 2.07, 95% CI, 1.03–4.14) [26]. Irfan et al. (2021) also reported higher odds of anxiety among participants that experienced a loss in income (AOR: 1.71, 95% CI, 1.34–2.17). Nonetheless, these findings contradict other studies in which family income was a protective factor for depression (AOR: 0.61, 95% CI, 0.44–0.84) [29], and being in low and middle-income groups were protective for stress (AOR:0.43, 95%CI, 0.26–0.71) [23]. Although poor perceived financial status influenced mental health negatively [23, 36], there is no strong evidence to demonstrate the association between both concepts.

### Social factors

Several social factors impacted mental health negatively, such as being a student [23], single or divorced; having more than three children [25], staying alone [32], being hospitalized for a surgical procedure [26], privacy at home, exercise [29], and social conflict [38]. In contrast, having good internet access [35], internet addiction [37], strong social support [25, 30], and long job service [36] were protective factors against mental health problems. Strong social support from family was also protective against stress among healthcare workers, especially among nurses (AOR: 0.91, 95% CI: 0.83, 0.99) [25]. Kalok et al. (2020) also documented that family support was protective against depression among university students (AOR: 0.35, 95% CI:0.14, 0.84) [30]. Nevertheless, the social factors findings are not rigorous due to sparse studies, limiting its generalizability to the Malaysian population.

### Academic factors

Studies conducted among junior [30, 37] and management university students [32] depicted that they have higher anxiety levels. These findings differ from a study, which postulated that postgraduate students have a higher risk for anxiety compared to undergraduate students [31]. A definite finding could not be supported given the limited data and a lack of substantial evidence.

### Psychological factors

Avoidant coping affected healthcare workers’ mental health negatively, whereas religious coping was protective against anxiety and stress [25, 36]. A higher risk of anxiety was reported among healthcare workers, especially doctors and nurses that employed avoidant coping (AOR: 1.25, 95% CI:1.15, 1.37 [36]. Meanwhile, healthcare workers that deployed religion to cope with the pandemic experience less anxiety and stress [25, 36]. A high happiness score was protective against stress and depression in healthcare workers [38]. This review highlighted the psychological factors that might play vital roles in predicting mental health issues among healthcare workers, but substantial evidence is lacking.

### COVID-related factors

Studies reporting the link between COVID-related factors and mental were included in this systematic review. Resultantly, positive perception of COVID-19 and the availability of its test facilities were protective against mental health [28, 31]. Meanwhile, a higher risk of suffering from mental issues was associated with the negative perception of susceptibility to COVID-19 and the severity of the virus [34, 39]. These findings reflect that COVID-related perceptions had a strong impact on mental health during the pandemic. Other COVID-related factors, such as the presence of symptoms [24], exposure to news [35], infected relatives or friends [29, 35], fear [25, 39], impact [39], and quarantine [25, 33] were positively associated with a greater risk for depression, anxiety, and stress across different populations.

## Discussion

The COVID-19 pandemic has adversely impacted the mental health of different populations worldwide. To date, this is the first systematic review to investigate the prevalence and factors associated with depression, anxiety, and stress in Malaysia during the COVID-19 pandemic. Data retrieved from 16 cross-sectional studies were analysed. The prevalence of mental health issues in Malaysia during the pandemic ranged from 14% to 81.7% for depression, 8.0% to 81.7% for anxiety, and 0.9% to 56.5% for stress, thus indicating the wide margin between the prevalence estimates. The prevalence of depression was highest among adult populations, whereas students recorded the highest prevalence of anxiety and stress during the pandemic. Given that only participants between 21 and 59 years were included in this review, the findings should be generalized with caution.

Previous National Health and Morbidity Survey (NHMS) revealed a decrease in the prevalence of depression from 29.2% in 2015 to 2.3% in 2019 due to differentials in screening modalities used [40, 41]. The prevalence increased during COVID-19 and ranged from 14.3% to 81.7%. Nevertheless, the trend of prevalence across the years might be influenced by the heterogeneity of depression screening tools used in NHMS 2015 (General Health Questionnaire-12), NHMS 2019 (Patient Health Questionnaire-9), and the current review (involving three different inventories). Similarly, the trends of anxiety increased during the pandemic with a prevalence between 8.0% and 81.7% relative to the pre-COVID estimates which ranged from 0.3% to 6.5% [19]. Meanwhile, the trend of stress remained the same before and during the pandemic. A previous systematic review reported a high prevalence of stress before COVID-19 at 56% among medical students in Malaysia [42]. Likewise, the present review found that university students experienced a high prevalence of stress at 56.5%. The trend of stress before and during the pandemic could not be further elucidated as only two studies focused on the prevalence of stress among university students. Although the global prevalence trends of mental health issue are rising [43–46], there is no empirical evidence to robustly compare the national trends of prevalence from pre-pandemic to pandemic period in general population or certain subgroup populations. Subgroup comparison between pre-pandemic and pandemic prevalence of the outcomes are limited due to different screening modalities. In adult population, PHQ-9 were used in NHMS 2019 [41] but only one study utilised identical screening tool for screening adult depression prevalence during the pandemic [29]. Prevalence of anxiety among university students from a large-scale national study in 2019 [47] is also incomparable due to limited study [47] using identical tools i.e., GAD-7. This review revealed that most studies used DASS-21 as a screening tool. It highlighted a major limitation which is inadequate studies with consistent screening practice. Nonetheless, this review posits that the high prevalence of mental health conditions during the pandemic were predominantly related to biological, financial, and COVID-related factors.

These aforementioned domains provide substantial evidence in justifying the factors associated with mental health issues. Among biological factors, age and gender were the most consistent variables associated with depression, anxiety, and stress. Age is a well-known risk factor for depression, anxiety, and stress in various populations. This review found six articles reported consistent findings regarding the association between age and mental health problems in adult populations and university students [23, 28–32]. In both groups, younger populations were at a higher risk for depression and anxiety. This finding coincides with the results from several systematic reviews [48–50]. The implementation of MCO during COVID-19 elevated anxiety levels among young university students due to the uncertainty about the effectiveness of online study platforms, apprehension of examinations, and future job employment [32]. Young students may be less mature in thinking and have low confidence and optimism about the pandemic [51]. Hence, they feel anxious in the transition from physical learning to online learning, and the completion of course tasks at home while considering how long the university will remain closed.

The findings from this systematic review align with other previous reviews in which women suffered from worse mental health outcomes in comparison to men during the pandemic [50, 52]. Numerous studies reported that Malaysian women have higher depression and anxiety levels during COVID-19 secondary to multiple contributing factors [23, 29, 32–34]. Females exhibited higher adverse psychological responses when addressing strong stressors [53]. Being quarantined with children due to the closure of schools and kinder gardens, compounded with office work at home may contribute to the worsening mental health status. Additionally, females may be less willing to seek professional help and ask for support due to mental health stigma, exacerbating pre-existing problems [54].

Financial factors play vital roles in predicting mental health status. Several studies conducted during the COVID-19 pandemic in Malaysia found that loss of family income elicited mental health issues across different populations [26, 34, 35]. This factor resonates similarly across other systematic reviews undertaken in different countries [48–50]. Family income was either reduced or completely lost as a consequence of the national lockdown implemented in Malaysia to curb the spread of the virus. These measures caused a major closure of wholesale and retail sectors, manufacturing, accommodation and food services, real estate, business, transportation, and construction due to the massive decline in the demand for goods and services [55]. Workers were laid off with minimal opportunity of being re-employed during COVID-19 due to limited work availabilities. The unprecedented job losses impaired mental well-being significantly [56].

Mental health status was significantly affected by the perception of COVID susceptibility, severity, and test unavailability [28, 31, 34, 39]. Individual perceptions were mainly driven by the information they possessed. Insufficient information regarding COVID prevention, treatment, and vaccination due to poor risk communication during the pandemic may elicit higher levels of mental health issues among the population [39]. Frequent changes in social protocols during different phases of the MCO, including re-lockdown and stricter interstate travel after lifting the interstate ban might signal to the population of the worsening COVID situation in Malaysia. These events were further exacerbated by the increasing trends of daily COVID-19 cases and the emergence of Delta and Omicron variants, thereby prompting the population to perceive COVID-19 severity and susceptibility negatively [31, 39]. The availability of testing facilities is now an issue due to the sudden rise in the number of cases, which has affected the access and cost of performing COVID-19 tests. Test unavailability precipitates worsening mental health status among Malaysians [28].

### Limitation of this review

This review is subjected to a few limitations. Only articles published in English were included, which might lead to publication bias since articles written in Malay (i.e., the national language) were not included in the search strategy. All the studies used a cross-sectional design, thereby limiting the causality between factors and the psychological outcomes. Most of the included studies utilized online data collection methods due to the MCO, which might explain the data paucity on geriatric populations. Likewise, there are limited mental health studies among children and teenage populations. The closure of primary and secondary schools during the pandemic might contribute to this event, thus limiting researchers’ access to participants and guardians during the lockdown. A meta-analysis was not conducted due to the heterogeneity of the included studies.

## Conclusion

High prevalences of mental health issues during a pandemic warrant for early mental health interventions. Differentials in screening practices limit comparison between pre-pandemic and pandemic prevalences. It is uncertain whether key at risk populations are vulnerable throughout or certain groups become even more susceptible to mental health issues during the pandemic. Hence, national policy on standardised screening practices using the best instrumental tool is highly recommended. Future research should zone in and conduct more rigorous screening among key at risk populations as well as evaluation assessments of interventions to start figuring out what works.

This review identified multiple factors associated with mental health issues during the COVID-19 pandemic. Specifically, age, gender, family income, and perception of COVID-19 demonstrated consistent findings. It unveiled a new unique factor during the pandemic i.e., COVID-related factors. These factors serve as a foundation for effective mental health intervention for national policymakers. Targeted interventions should be focused on specific population groups with effective risk communication intervention to improve COVID-19 perception among Malaysians. Future research on COVID-related factors should be explored on.

## Supporting information

S1 Checklist PRISMA 2020 checklist. (DOCX)
